# Low fruit and vegetable intake in children: a dietary stressor raising renal ammonium and adrenal cortisol secretion

**DOI:** 10.1007/s00424-026-03162-3

**Published:** 2026-03-23

**Authors:** Thomas Remer, Yifan Hua, Jonas Esche, Lijie Shi, Michaela F. Hartmann, Stefan A. Wudy

**Affiliations:** 1 DONALD Study Center, Nutritional Epidemiology, Institute of Nutrition and Food Science, University of Bonn, Heinstück 11, Dortmund, 44225 Germany; 2 Formerly at DONALD Study Center, Nutritional Epidemiology, Institute of Nutrition and Food Science, University of Bonn, Dortmund, Germany; 3 Laboratory for Translational Hormone Analytics, Steroid Research & Mass Spectrometry Unit, Pediatric Endocrinology & Diabetology, Center of Child and Adolescent Medicine, Justus Liebig University, Giessen, Germany

**Keywords:** Ammonium excretion, Cortisol, Dietary acid load, Fruit and vegetable intake, Glucocorticoid secretion, Net acid excretion, Renal stress

## Abstract

Elimination of excess acid loads in metabolic acidosis requires stimulation of ammoniagenesis. Cortisol is critical to increase proximal tubular cells’ ammonia production. We aimed to specifically examine whether children’s low fruit and vegetable (FV) intake, which goes along with an increased dietary acid load, may raise cortisol secretion and whether glucocorticoids relevantly associate with ammonium secretion. Healthy 6–10 years old children (participants of the DONALD study, Dortmund, Germany) with a comparable protein intake and either a low or a high FV-intake were examined in a quasi-experimental design. Urinary 24-h ammonium excretion was analyzed as part of urinary acid-base measurements. Major glucocorticoid metabolites were quantified along with aldosterone precursors in children’s 24-h urine samples by gas chromatography–mass spectrometry to assess adrenal cortisol secretion and aldosterone status. Liquid–chromatography–mass spectrometry was used to quantify urinary free cortisol (UFF) and cortisone. Average FV-intake of children with low compared to high FV-intake was 183 g/d (25th -75th percentile, 129–233 g/d) and 570 g/d (25th -75th percentile, 468–707 g/d), respectively. Both groups’ average protein intake amounted to around 50 g/d. After adjustment for relevant confounders, children with low FV-intake exhibited higher excretion rates of UFF (*P* = 0.002), cortisone (*P* = 0.07), potentially bioactive free glucocorticoids (sum of UFF and cortisone) (*P* = 0.04), 20α-dihydrocortisol (*P* = 0.02), C21-metabolite sum reflecting cortisol secretion (*P* = 0.008), and ammonium (*P* < 0.0001). Concurrently with these children’s lower potassium intake, excretions of the aldosterone precursors tetrahydro-11-dehydrocorticosterone and tetrahydrocorticosterone were reduced (*P* ≤ 0.02). Urinary ammonium excretion associated closely with UFF (*P* < 0.0001), cortisone (*P* < 0.0001), and 20α-dihydrocortisol (*P* = 0.0003). Our quasi-experimental study indicates the importance of an appropriately high FV-intake in childhood to reduce acid-load-inducible cortisol-increases that are strongly associated with renal ammonium production and may have the potential to adversely affect long-term health.

**Supplementary Information:**

The online version contains supplementary material available at 10.1007/s00424-026-03162-3.

## Introduction

Glucocorticoids are vitally important hormones essentially involved in a vast number of metabolic processes of which one is the stimulation of ammonia production in the kidney. Ammonia is essential to buffer and renally excrete excess fixed acid loads. To attain this kidney health-relevant buffering, several physiological metabolic steps are required that all involve glucocorticoid activity. Glucocorticoids are critical for (1) the induction of skeletal muscle catabolism and release of glutamine, i.e., the primary substrate for renal ammonia production, (2) the renal cortical activation of the major glutamine uptake transporter of proximal tubule cells, and (3) the increase in proximal tubule ammonia generation itself and tubular cells’ ammonia secretion via the sodium/proton exchanger NHE3 [1].

In accordance herewith, several human studies found that correcting metabolic acidotic states and accordingly reducing renal ammonia secretion via oral alkalization or growth hormone administration reduces urinary excreted and/or circulating glucocorticoids [2–4].

In a previous study that examined hormonal implications of a high net endogenous acid production in children, we could show that the potential renal acid load (PRAL) and the gold standard measurement renal net acid excretion (NAE) analyzed in 24-h urine samples are both significantly positively related with adrenal cortisol secretion and the indicators of potentially bioactive free glucocorticoids urinary free cortisol (UFF) and free cortisone (UFE) [5].

As in the long run even only modestly increased cortisol levels can harmfully affect metabolic [6], cardiovascular [7], mental [8], and bone health [9], we aimed to specifically examine whether the reduced alkalizing potential of a low fruit and vegetable (FV) intake ingested along with a common moderately high protein intake may already inversely influence adrenal cortisol secretion of children and whether their glucocorticoid excretion may associate with renal ammonia production.

## Materials and methods

### Study population

All healthy children examined were participants of the DOrtmund Nutritional and Anthropometric Longitudinally Designed (DONALD) Study, Dortmund, Germany. The DONALD study aims to examine the long-term complex relations between dietary intake, metabolism, and growth in healthy children and adolescents until their adulthood. Usually, during the first 2 y of life, children along with one or both parents visit the DONALD study quarterly or half-yearly, and then from 3 to 4 y onward, a yearly 24-h urine collection is scheduled in addition to anthropometric measurements, provision of a 3-d weighed dietary record, medical examinations, and interviews on lifestyles [10]. All assessments and examinations are conducted with parental and grown-up children’s written consent and are performed in accordance with the guidelines of the Declaration of Helsinki. The study protocol has been approved by the ethics committee of the University of Bonn, Germany (approval Nos. 098/06 and 185/20).

**Fig. 1:**
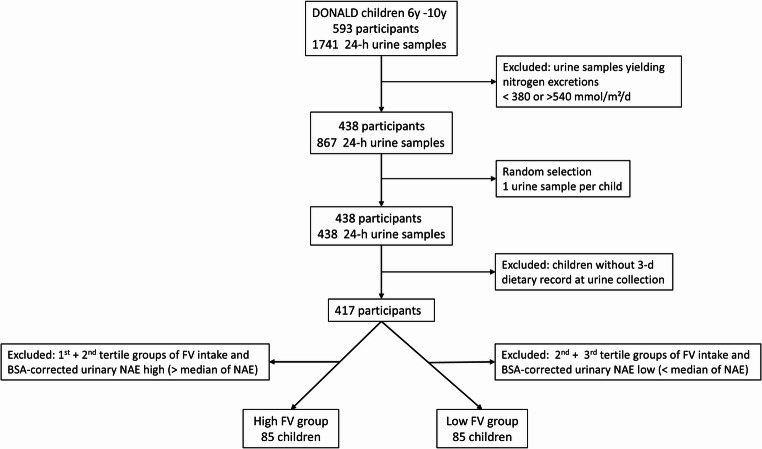
Flow chart summarizing inclusion steps and exclusion criteria. The lower exclusion level of 380 mmol nitrogen per day reflects the arithmetic mean of the recommended dietary allowances for protein for 3–8 and 9–14 years old children when estimating total nitrogen excretion from protein intake [70] under the assumption of 20% etrarenal losses of nitrogen

From the participating healthy children of the DONALD study who had provided at least one plausible and complete 24-hour urine collection and in whom anthropometric measurements to assess fat free mass (FFM) [11] were available, the subsample of 6- to 10-year-olds (*n* = 593) with a total of 1741 24-h urine collections was retrospectively selected. To ensure appropriate and comparable protein intake, only urine collections from children with a 24-h nitrogen excretion in the range from 380 mmol/d (5th percentile of nitrogen excretion of healthy children [12]) to 540 mmol/d (mean nitrogen excretion of healthy children with sufficient protein intake [13]) were included (Fig. 1, *n* = 867). Based on this sample, comprising children with more than one urine collection, a random selection was conducted to obtain one 24-h urine sample per child (*n* = 438). In 417 of these children, a 3-d dietary record was available at the time of urine collection. To avoid extra high endogenous acid loads (of not further specified origin) in children with high FV-intake, those children out of the 417, lying within the highest tertile of FV-intake and at the same time in the bottom half of body surface area (BSA)-corrected 24-h NAE, were assigned to the high-FV group (*n* = 85). Correspondingly, those in the lowest tertile of FV-intake and in the upper half BSA-corrected NAE were placed into the group low-FV (*n* = 85).

### Dietary measurements

Dietary intake was assessed by 3-d weighed dietary records according to instructions by trained dieticians. All consumed foods, beverages, and leftovers were weighed to the nearest 1 g using electronic food scales and recorded for 3 consecutive days by parents or by the adolescent participants themselves. Dietitians checked the dietary records for accuracy and completeness before data entry into and calculation of nutrient intake through the continuously updated in-house nutrient database LEBTAB [14]. Individual means of energy, FV, and nutrient intake calculated from the 3-day dietary records were used in analyses. The FV-intake was determined using the following formula: fruit intake (g/d) + vegetable intake (g/d) + juice intake (g/d), if a 100% fruit or a 100% vegetable juice was consumed [15].

### Urinary measurements

All 24-h urine collections were performed at home in preservative-free, Extran-cleaned (Extran, MA03; Merck) 1-L plastic containers and stored at ≤ −18 °C until being thawed for analyses. The 24-h urine collections were considered as plausible and complete when body weight-related 24-h creatinine excretion rate was ≥ 0.1 mmol/kg/d and samples were not reported to contain improper micturition. Urinary creatinine was analyzed with a creatinine analyzer (Beckmann-2; Beckman Instruments, Inc.) using the kinetic Jaffé procedure.

Total urinary nitrogen was measured by Kjeldahl technique (Büchi 430 Digestor and Büchi Distillation unit B-324; Büchi Labortechnik, Flawil, Switzerland). Organic acid anions were measured according to the Van Slyke and Palmer titration method [16, 17]. The 24-h urinary anions chloride, phosphate, and sulfate were quantified by using Dionex 2000i/SP ion chromatography with an ion Pac AS4A column (Dionex GmbH), and cations potassium, magnesium, calcium, and sodium by flame atomic absorption spectrometry (Perkin Elmer 1100 Spectrometer; Perkin Elmer). Urinary citrate concentrations were analyzed following enzymatic conversion of citrate via oxaloacetate to L-lactate using a commercial citrate assay kit (Boehringer Mannheim, Germany). Daily acid load was assessed by measurement of 24-h renal net acid excretion (NAE) and urinary potential renal acid load (uPRAL). NAE was determined using the following formula: NAE = Titrable acids + NH_4_^+^ - HCO_3_^−^; and uPRAL [18] as follows: uPRAL = [chloride (mmol/d) + sulfate (mmol/d) * 2 + phosphate (mmol/d) * 1.8] - [sodium (mmol/d) + potassium (mmol/d) + magnesium (mmol/d) * 2 + calcium (mmol/d) * 2]. Net acid excretion capacity (NAEC) was calculated as residuals of NAE on urine pH [19].

Daily excretion rates (µg/d) of total urinary steroids were analysed by targeted GC-MS urinary steroid metabolome analysis of 24-h urine samples as described previously [20, 21]. In brief, conjugated and unconjugated steroids were extracted by solid phase extraction followed by enzymatic hydrolysis. After re-extraction, the steroid extract was derivatised to methyloxime-trimethylsilyl-ethers and the samples thereafter subjected to GC-MS analysis with the MS operated in selected ion monitoring mode. To assess overall daily GC secretion, the following 7 quantitatively most important urinary GC metabolites were measured and summed (ΣC21): tetrahydrocortisone, tetrahydrocortisol, 5a-tetrahydrocortisol, a-cortolone, b-cortolone, a-cortol, and b-cortol [21]. ΣC21 includes around 80% of adrenal glands total cortisol secretion [22]. 20α-dihydrocortisol, tetrahydro-11-dehydrocorticosterone, and tetrahydrocorticosterone were also quantified by GC-MS. Urinary free cortisol (UFF) and urinary free cortisone (UFE) were measured by liquid chromatography–tandem mass spectrometry analysis [23].

### Statistical analysis

All statistical analyses were performed using SAS procedures (SAS Institute Inc., Cary, NC, USA; version 9.4). A significant effect was considered if *P* < 0.05. All variables were checked for normal distribution using the Shapiro-Wilk test and Q-Q plot.

General characteristics of the study sample are shown as mean ± SD for normally distributed or median (25th, 75th percentile) for non-normally distributed parameters. Differences in basic characteristics of the study population between low- and high-FV group were tested with unpaired *t*-test for normally distributed variables and nonparametric Wilcoxon test for non-normally distributed variables. Non-normally distributed variables were log-transformed to approximate a normal distribution.

The independent associations of FV-intake with glucocorticoid outcome variables (i.e., UFF, UFE, sum of UFF and UFE, 20α-dihydrocortisol and ΣC21) were tested separately using multilinear regression analyses (PROC GLM in SAS). All regression models were run with logarithmized outcomes and covariates. Urinary concentrations of the glucocorticoid metabolite 20α-dihydrocortisol were found to lie below the limits of detection (LOD) in 32 children. These children’s 20α-dihydrocortisol concentrations were set at a proposed low-range level equaling to the metabolite’s LOD (i.e., 12.5 µg/L) divided by the square root of 2 [24, 25]. Since no sex interactions were observed, regressions were done for the total sample of 170 children. FV-intake was included as categorical variable (low- vs. high-FV-intake). PROC GLM regressions were also conducted for the assessment of the relationship between ammonium excretion and glucocorticoid metabolites, as well as between acid-base biomarkers (i.e., NAEC and citrate) and the muscularity indicator fat-free mass (FFM). Potential covariates were tested separately using stepwise regression before being included in the final statistical model. Covariates were included if they modified the association between FV-intake predictor and the glucocorticoid outcomes (i.e., changes of the β coefficients > 10%); or f they improved the explained variability of models. Variables were retained in the model if they met the predetermined level of *P* ≤ 0.2. Potential confounders considered were sex, duration of urine storage, urinary nitrogen, urinary organic acid, and urinary sodium.

## Results

Children’s anthropometric, dietary, and renal excretion characteristics are specified in Table 1. Anthropometric data, energy, total protein intake, and animal protein percentage, 24-h urine volume, BSA-corrected nitrogen excretion, and excretion rates of urea, creatinine, and sodium did not significantly differ between children with low and high FV-intake. In the latter group average FV and protein intake was 570 and 49 g/d, respectively vs. 183 and 51 g/d in low FV consumers.

**Table 1:** Characteristics of the study population (*n* = 170)a

	Low-FV group	High-FV group
	(*n* = 85, 41 boys)	(*n* = 85, 42 boys)
Anthropometric measurements		
Age (yr)	9.0 (7.0, 9.9)	7.9 (7.0, 9.0)
Height (cm)	132.7 ± 10.4	129.7 ± 9.7
Weight (kg)	28 (24.2, 35.1)	26.4 (23.0, 31.3)
Fat free mass (kg)	23.8 (20.5, 27.6)	22.2 (19.7, 25.1)
BMI (kg/m^2^)	16.7 ± 2.4	16.4 ± 1.8
BSA (m^2^)	1.05 ± 0.17	1.00 ± 0.15
**3-d dietary data**		
Total energy (ecal/d)	1558.4 (1376.3, 1749.0)	1633.5 (1405.8, 1823.9)
Total protein (g/d)	51.4 (43.6, 56.2)	48.9 (42.2, 55.9)
Animal protein proportion (%)	64.4 ± 7.1	61.7 ± 9.2
Total protein (g/kg/d)	1.78 (1.49, 2.01)	1.77 (1.60, 2.03)
FV intake (g/d)^**b^	182.8 (128.7, 233.0)	569.7 (468.4, 707.1)
Potassium (mg/d)^**^	1928.2 (1666.6, 2213.4)	2395.2 (2093.7, 2841.1)
Magnesium (mg/d)^**^	202.9 (181.1, 233.4)	241.3 (205.8, 279.5)
**24-h urinary nonhormonal data**		
Duration of storage [urine] (yr)	18.0 (14.0, 22.0)	15.0 (11.0, 20.0)
Urine volume (ml)	555.2 (408.5, 676.9)	754.8 (534.4, 963.5)
Urine pH^**^	5.8 ± 0.4	6.7 ± 0.2
NAE (mEq/d)^**^	44.2 (36.7, 49.0)	22.0 (16.7, 26.3)
NAE (mEq/d/1.73 m^2^)^**^	72.3 (65.6, 80.6)	37.9 (30.2, 46.6)
Ammonium (mmol/d)^**^	27.9 (23.9, 31.6)	20.5 (18.2, 24.5)
Ammonium (mmol/d/1.73 m^2^)^**^	46.1 (40.4, 52.0)	37.3 (33.0, 41.1)
Titratable acidity (mEq/d)^**^	15.7 (13.3, 20.5)	7.0 (5.0, 9.2)
Bicarbonate (mEq/d)^**^	0.0 (0.0, 0.0)	6.8 (5.0, 8.7)
Citrate (mmol/d)^**^	1.46 (0.97, 1.90)	1.92 (1.45, 2.46)
uPRAL (mEq/d)^**^	16.4 (9.9, 20.7)	−7.6 (−13.5, −0.3)
Urinary organic acids (mEq/d)^*^	24.9 (21.5, 28.5)	27.6 (23.8, 31.1)
Nitrogen (mmol/d)^*^	467.6 (412.9, 536.0)	434.7 (391.9, 504.8)
Nitrogen (mmol/d/1.73 m^2^)	802.1 (721.0, 863.3)	751.6 (705.3, 834.6)
Urea (mmol/d)	208.4 (184.8, 242.7)	196.9 (175.9, 234.1)
Creatinine (mmol/d)	4.7 (3.7, 5.6)	4.4 (3.7, 5.1)
Sodium (mmol/d)	72.9 (57.9, 94.6)	74.7 (58.2, 91.0)
Potassium (mmol/d)^**^	34.4 (26.8, 41.6)	46.5 (39.0, 52.3)
**24-h urinary glucocorticoid data**		
Urinary free cortisol (µg/d)	7.7 (5.6, 11.3)	6.9 (5.6, 9.0)
Urinary free cortisone (µg/d)	16.2 (12.6, 21.6)	16.9 (12.9, 22.1)
Urinary free cortisol + cortisone (µg/d)	23.4 (18.9, 34.1)	23.8 (19.8, 29.4)
Urinary 20α-dihydrocortisol (µg/d)^*c^	16.2 (12.0, 20.8)	12.8 (9.5, 18.7)
ΣC21 (µg/d)	2667.8 (2120.6, 3317.2)	2354.6 (1972.3, 2984.7)
Tetrahydro-11-dehydrocorticosterone (µg/d)^*^	43.0 (27.2, 60.9)	54.7 (36.7, 80.3)
Tetrahydrocorticosterone (µg/d)^*^	27.3 (20.4, 38.4)	40.7 (27.9, 56.9)

*BMI* body mass index, *BSA* body surface area, *FV intake* fruits and vegetable intake, *NAE* renal net acid excretion, *uPRAL* urinary potential renal acid load, *ΣC21* sum of the 7 quantitatively most important urinary glucocorticoid metabolites, representing total daily adrenal cortisol (overall glucocorticoid) secretion

^a^Values are presented as mean ± SD if normally distributed or as median (25th, 75th percentiles) if non-normally distributed

^b^Average ingested amounts of FV corresponded to approximately 2.3 servings in the low FV group and 7 servings in the high FV group (based on a typical adult FV serving of 80 g)

^c^Missing values of 20α-dihydrocortisol were imputed using its limit of detection (LOD)

^*^P < 0.05

^**^P < 0.0001. Differences were tested with unpaired *t*-tests for normally distributed variables and nonparametric Wilcoxon tests for nonnormally distributed variables

**Fig. 2:**
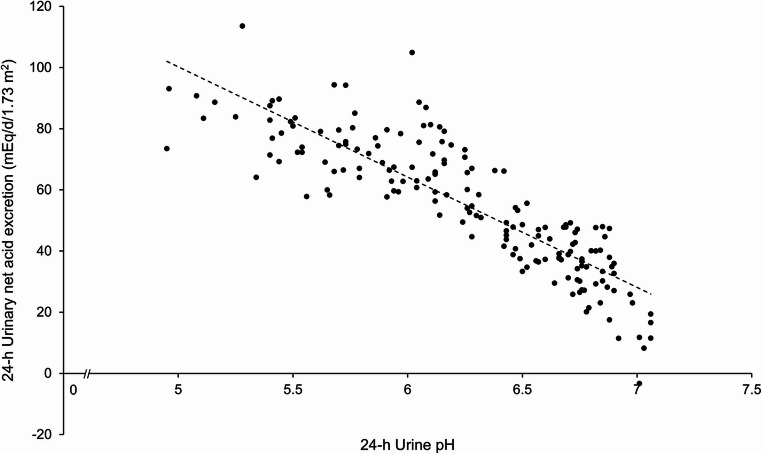
Relationship between body surface area-corrected renal net acid excretion (NAE) per day and 24-h urinary pH in the two groups of 6–10 years old children consuming either high FV or low FV diets

In accordance with the respective dietary habits the urinary potassium, magnesium, organic acid, bicarbonate, and citrate excretion as well as the 24-h urine pH were lower and NAE, titratable acidity, ammonium, and urinary PRAL higher in the low-FV group. In 20% of the children consuming low FV, but in none eating high amounts, 24-h urinary pH values below 5.5 were observed (*n* = 17, Fig. 2).

Among the renally excreted adrenocortical hormone metabolites examined, only three differed significantly between groups. Tetrahydro-11-dehydrocorticosterone and tetrahydrocorticosterone were significantly lower and 20α-dihydrocortisol significantly higher in low-FV eaters (Table 1). When FFM was tentatively examined as an outcome reflecting children’s muscularity status, both NAEC (*P =* 0.02) and renal 24-h citrate excretion (*P* = 0.005) associated positively with this marker of muscularity after adjusting for sex, fat mass, height, and nitrogen excretion (Table 2).

**Table 2:** Relation of two rather sustained indicators of acid-base status namely renal net acid excretion capacity (NAEC) and renal 24-h citrate excretion with the muscularity marker fat free mass

	β-values	95% CI	*P*	*R* ^2^_model_
Indicator 1				
NAEC^a^	0.049	0.0078, 0.090	0.02	0.89
Sex	0.95	0.44, 1.46	0.0003	
Fat mass	0.24	0.13, 0.34	< 0.0001	
Height	0.32	0.28, 0.35	< 0.0001	
Nitrogen excretion	6.58	0.70, 12.5	0.03	
**Indicator 2**				
Citrate	0.42	0.13, 0.72	0.005	0.89
Sex	0.96	0.46, 1.46	0.0002	
Fat mass	0.23	0.13, 0.33	< 0.0001	
Height	0.31	0.27, 0.34	< 0.0001	
Nitrogen excretion	9.92	4.81, 15.04	0.0002	
R^2^_model_, total explained variance				

^a^NAEC, calculated as residuals of measured 24-h net acid excretion (NAE) on 24-h urine pH, provides an estimate of the kidney’s ability to excrete acid loads

**Fig. 3:**
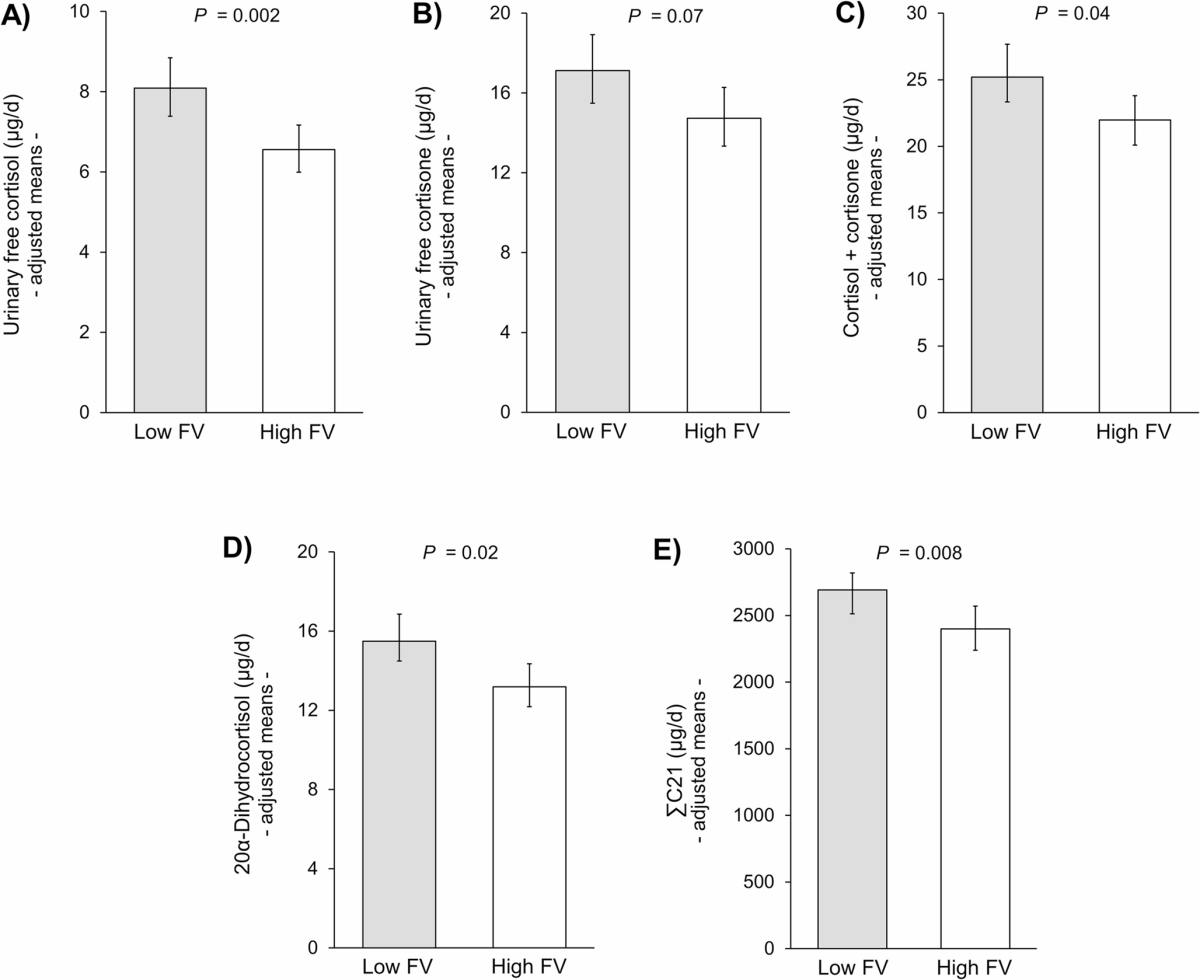
Urinary free cortisol, cortisone, potentially bioactive free glucocorticoids (urinary free cortisol + cortisone), 20α-dihydrocortisol, and cortisol secretion (ΣC21) in children with low (*n* = 85) and high (*n* = 85) fruit and vegetable (FV) intake. Data are anti-log-transformed least-squares means with 95% confidence intervals derived from multiple linear regressions and FV intake as categorical variable. Missing values of 20α-dihydrocortisol were imputed using its limit of detection (LOD). All models were adjusted for sex and urinary organic acid. For the outcome urinary free cortisol, the regression model was additionally adjusted for urinary sodium and sample storage duration; for urinary free cortisone and potentially bioactive free glucocorticoids additionally for urinary sodium, storage duration, and urinary nitrogen; and for 20α-dihydrocortisol and ΣC21 additionally for urinary nitrogen

After adjustment for relevant covariates and confounders, children with high FV-intake exhibited lower UFF (*P* = 0.002), almost significantly lower UFE (*P* = 0.07), lower potentially bioactive free glucocorticoids (sum of UFF and UFE) (*P* = 0.04), lower 20α-dihydrocortisol (*P* = 0.02), and a lower cortisol secretion, assessed via ΣC21 (*P* = 0.008) (Fig. 3). By contrast, the initially already higher 24-h excretion rates of the aldosterone precursors tetrahydro-11-dehydrocorticosterone and tetrahydrocorticosterone of high-FV eaters (Table 1) remained significantly elevated (*P* = 0.02 and *P* = 0.01, respectively) after adjustment for sex, organic acids, and ΣC21 (data not shown). Adjustment for the latter was done due to the clear stimulatory effect of ACTH not only on ΣC21, but also on aldosterone secretion [26, 27].

**Fig. 4:**
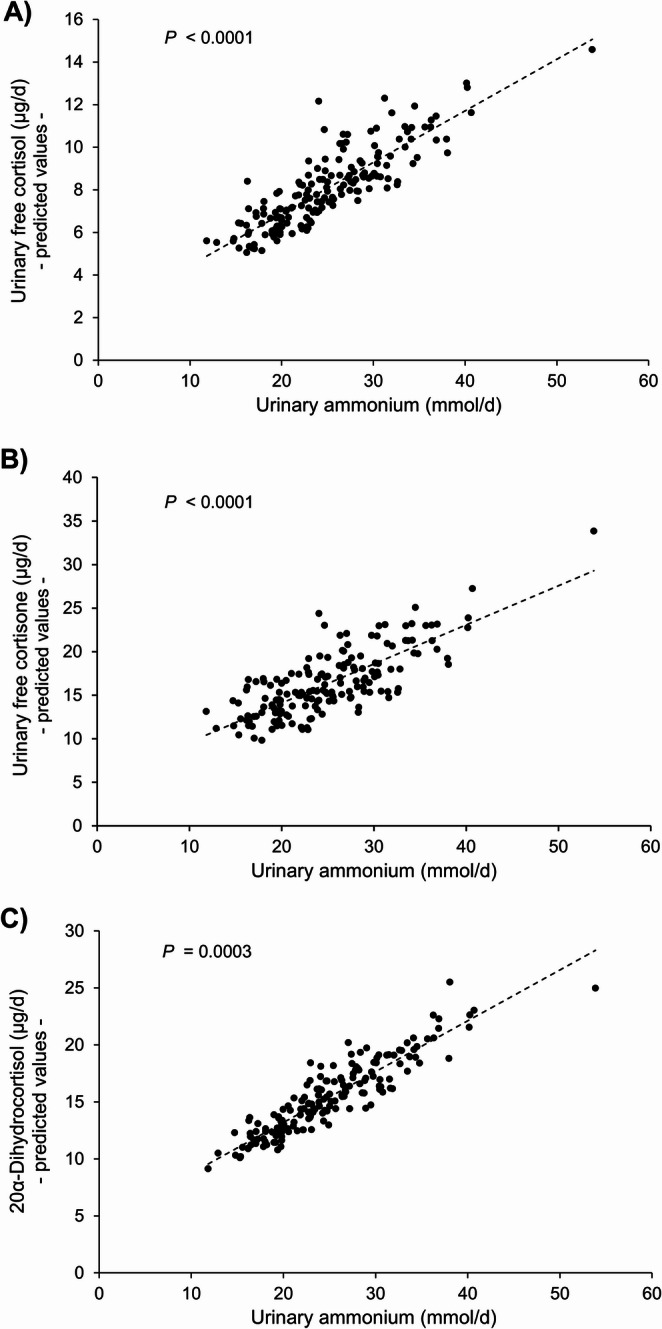
Relation of 24-h urinary ammonium excretion with adjusted urinary 24-h excretion rates of glucocorticoids. Shown are predicted values of **(A)** urinary free cortisol (UFF) adjusted for its relationship with sodium excretion, **(B)** urinary free cortisone (UFE) accordingly adjusted for sodium excretion and storage duration, and **(C)** 20a-dihydrocortisol adjusted for body surface area (BSA). All adjusted covariates had been specifically identified in the multilinear regressions given in Table 3. Of the overall explained variations R^2^_model_ of 0.23, 0.21, and 0.21 in the regression models for UFF, UFE, and 20α-dihydrocortisol, distinct proportions of almost 50%, 40%, and 30% were due to the predictor ammonium, explaining at least in part the very good data fitting of the predicted values in panels A-C. Above % proportions of ammonium were deduced from the semi-partial explained variances (R^2^_semi−partial_) of 0.11, 0.08, and 0.06 for UFF, UFE, and 20α-dihydrocortisol, respectively (Table 3)

Furthermore, UFF (*P* < 0.0001), UFE (*P* < 0.0001), and 20α-dihydrocortisol (*P* = 0.0003) were highly significantly related to 24-h urinary ammonium excretion, whereas ΣC21 was not and the sum of UFF and UFE just exhibited a trend (Table 3). Figure 4 illustrates the relationships between ammonium excretion and the above significant glucocorticoids UFF, UFE, and 20α-dihydrocortisol after adjustment for these hormones’ associations with the significant covariates identified in Table 3.

**Table 3:** Unadjusted^a^ and adjusted^b^ associations of 24-h urinary glucocorticoid metabolites with 24-h urinary ammonium excretion

	β-values	95% CI	*P*	*R* ^2^_model_
**Urinary free cortisol**				
Unadjusted	0.029	0.019, 0.039	< 0.0001	0.16
Adjusted^c^	0.024	0.014, 0.034	< 0.0001	0.23
**Urinary free cortisone**				
Unadjusted	0.026	0.015, 0.036	< 0.0001	0.12
Adjusted^d^	0.021	0.011. 0.032	< 0.0001	0.21
**Urinary free cortisol + cortisone**				
Unadjusted	0.026	0.017, 0.035	< 0.0001	0.16
Adjusted^e^	0.012	−0.932, 0.024	0.052	0.30
**Urinary 20α-dihydrocortisol** ^f^				
Unadjusted	0.012	0.008, 0.016	< 0.0001	0.18
Adjusted^g^	0.0086	0.0039, 0.013	0.0003	0.21
**ΣC21**				
Unadjusted	0.010	0.007, 0.013	< 0.0001	0.21
Adjusted^h^	0.0012	−0.0019, 0.0043	0.4	0.52

ΣC21, sum of the 7 quantitatively most important urinary glucocorticoid metabolites, representing total daily adrenal cortisol secretion. R^2^_model_, total explained variance.

^a^Simple regression of glucocorticoids on ammonium.

^b^Multilinear regression analyses testing the following 6 covariates for each of the given glucocorticoids: sex, body surface area, urine storage duration, urinary nitrogen, urinary organic acids, and urinary sodium. Covariates remaining in the final adjusted models^c−e, g,h^ were all significant. Not included covariates showed *P* > 0.2.

^c^Urinary cortisol adjusted for 24-h urinary sodium.

^d^Urinary cortisone adjusted for 24-h urinary sodium and storage duration.

^e^Urinary free cortisol +cortisone adjusted for 24-h urinary sodium, storage duration, organic acid, and urine pH.

^f^Missing values of 20α-dihydrocortisol were imputed using its limit of detection (LOD).

^g^20α-dihydrocortisol adjusted for body surface area.

^h^ΣC21 adjusted for 24-h urinary sodium, organic acid, nitrogen, and body surface area.

^c, d,g^Semi-partial R^2^ values (R^2^_semi−partial_) of ammonium for the significant cortisol, cortisone, and 20α-dihydrocortisol were 0.11, 0.08, and 0.06, respectively. R^2^_semi−partial_ indicates the proportion (11%, 8%, 6%) of the total variance of the respective hormones which is explainable exclusively by ammonium excretion, independent of the other significant covariates

UFF, UFE, and 20α-dihydrocortisol were further examined in relation to the amount of FV consumed. A significant inverse association with FV was ascertained for UFF, an almost significant inverse association for 20α-dihydrocortisol, and no relationship for UFE (Supplemental Table 1). The inverse associations of UFF and 20α-dihydrocortisol were no longer detectable when the respective relationships were additionally controlled for ammonium excretion underscoring ammonium’s role as physiological mediator of the UFF and 20α-dihydrocortisol variations.

## Discussion

Metabolic acidosis and high acid loads to the body have been demonstrated to raise glucocorticoid secretion in animals and humans [3, 28–30]. Correcting metabolic acidotic states via alkalizing measures accordingly reduces glucocorticoid levels [2, 3]. The present study is the first to show that high FV-intake’s low PRAL-inducible reduction of ammonium production can be capable to significantly attenuate potentially bioactive free glucocorticoids and adrenal cortisol secretion in children on rather protein-rich diets which are common not only in western countries [31]. These findings may be of particular relevance with regard to the well documented potentially disease-causing long-term effects of even only moderately elevated cortisol levels on insulin-glucose homeostasis [32], cardiometabolic [6, 7, 33] and mental health [8, 34], and bone stability [9, 35]. Apart from such subclinical glucocorticoid-stress-triggered health risks that may potentially also evolve in the long-term from the high dietary acid loads of a low FV-intake, an additional kidney-stressor, i.e. the increased ammonium production, will be present due to the lacking acid-neutralizing potential of low-FV diets.

In principle, renal ammoniagenesis is an essential physiological process ensuring the buffering of excess protons that need to be renally excreted, thus preserving basal bicarbonate regeneration, i.e. the body’s central blood buffer system. Increases in circulating cortisol are required to raise ammonium production [36, 37] and thus are necessarily involved in raising renal buffering capacity. By contrast, insufficient ammonium production due to deficient corticosteroid secretion goes along with metabolic acidosis. Maintenance of adequate renal ammonia metabolism is thus protective against acidosis. However, clearly elevated intrarenal ammonia concentrations can – on the other hand – exert detrimental effects on kidney function, i.e., induce tubulointerstitial inflammation processes, that are mediated via activation of the alternative complement pathway [38–40].

Augmented intrarenal levels of ammonia may activate the proximal tubular cells’ complement cascade leading to tubular cell activation, cell injury, and release of proinflammatory cytokines [41], which eventually contribute to progressive tubulointerstitial damage [41, 42]. In line herewith two recent studies of our group could show that a habitual high ammonium excretion – still in the normal range in healthy children – associates with the circulating renal tubular injury marker interleukin-18 [43] and also with albuminuria [44] later in adulthood.

After review of the literature and according to our current knowledge, the clear relationships between renal ammonium excretion and urinary excretion rates of free cortisol, free cortisone, and 20α-dihydrocortisol – as identified in the present quasi-experimental study in healthy children (Fig. 4) – have not been described so far, neither in humans nor in animals. This is unexpected given that it has been appreciated for decades that glucocorticoids are essentially required for proximal tubular cells’ ammonia generation and that their deficiency impairs ammonium excretion [1, 45, 46]. An additional explanation for the perhaps surprisingly strong association of glucocorticoids with ammonium production can be seen in the fact that under acidotic conditions circulating corticosteroid-binding globulin’s binding affinity for cortisol falls [47] so that cortisol delivery to organs, tissues, and renal cellular structures via blood capillary systems with lower pH and buffer capacity increases.

Summing up these findings, it can be concluded that in case of an insufficient alkalizing nutrient intake, i.e. in case of long-term low FV and concomitantly high protein intake, two potential stressors may be relevantly boosted: a specific intrarenal one, namely ammonium production, and a cardiometabolism-, mental health-, and bone health-related one, namely the glucocorticoid dyad cortisol & cortisone along with metabolites. Whether correspondingly elevated glucocorticoid levels may contribute to a potentially reduced anabolism in children with a reduced FFM and a long-term unfavorable acid-base status (e.g., a lower NAEC and a lower citrate excretion, Table 2) needs to be examined in much more detail in future.

The fact that the marker of cortisol secretion ΣC21, shown to be higher with the low FV-intake, did not significantly correlate with ammonium excretion may be due to metabolic disparities in cortisol clearance rates and the corresponding hypothalamic-pituitary-adrenal (HPA) axis’ responses. Both, cortisol clearance rates and HPA axis activities, can be influenced by dietary consumption of a number of plant-based organic acids and flavonoids [48–52]. Cortisol secretion (ΣC21), i.e., HPA axis activity needs not necessarily be elevated if metabolic cortisol action is increased in various cell systems and tissues [22, 53]. In this regard, it is notable that the urinary glucocorticoid metabolite 20α-dihydrocortisol is regarded to reflect glucocorticoid activity better than the commonly measured marker urinary free cortisol for glucocorticoid status [54]. All in all, the significant relationships of all three glucocorticoids UFF, UFE and 20α-dihydrocortisol underscores the importance of the glucocorticoid-ammoniagenesis relationship for balancing acid-base variations already in the normal physiological range.

Evidently, the present findings may also be seen as important with regard to the worldwide observed associations of low food security and low FV-intake in children and adults [31, 55–57]. Low food security is particularly prevalent in low income families and persons of low socioeconomic status, but it is also increasingly observed in high-income economies [56]. Thus, a preventable potentially systemically active stressor may additionally adversely affect children’s health. This is the low FV-intake in daily meals, going along not only with a low availability of major anti-inflammatory and antioxidative plant-based food compounds [58], but also with potentially raised glucocorticoid levels and increased renal ammonia production. For certain, this may not only affect children living in low-income circumstances, who probably already suffer from psychosocial and/or environmental stressors, but also those of higher-income parents.

Food insecurity in early childhood has been shown to associate inversely with young adults’ cardiovascular health as assessed through the ‘American Heart Association (AHA) Life’s Essential 8 score’ [59]. Interestingly, most of the 8 health and behavior factors defining the latter score, i.e., diet quality, blood lipids [6, 60, 61], blood pressure [6, 60, 61], blood glucose [6, 60, 61], body mass index [62, 63], and sleep health [61, 64] are also adversely affected by subclinical mild cortisol secretion. These findings along with our study results suggest that a varying glucocorticoid stress activity may be one important mechanism underlying the much-discussed relationship [65, 66] between habitual FV consumption and cardiometabolic health, besides further mechanisms as for example the anti-inflammatory and antioxidative effects of flavonoids.

Our study has several strengths. First, it examines healthy free-living children on their normal diets in a quasi-experimental design allowing causal inferences to some substantial degree, despite the principally observational nature of the data. Children’s dietary protein intake closely corresponded to the median protein intake of preadolescent boys and girls as examined in the previous representative German KiGGS survey [67] and it was consistently above the recommended dietary allowances as is particularly ascertainable by comparing with previously published data [68]. Appropriateness of protein intake was strictly biomarker-based controlled using children’s 24-h urinary nitrogen excretion as the exclusive primary inclusion criterion followed by an intra-individual random sample selection to ensure the specific cross-sectional grouping design. Second, allocation of children to the respective high- or low-FV group was based on detailed 3-day weighed dietary records conducted during days including the 24-h urine collection. Third, the use of GC-MS and LC-MS/MS for the corticosteroid analytics in 24-h urine samples enabled us to differentiate between potential bioactive metabolites on the one hand and adrenal glands’ secretion of cortisol and to some extent also of aldosterone on the other hand. Fourth, renal ammonium production could be assessed as part of the NAE measurement, which is the gold standard for examining net endogenous acid production.

There are also limitations to be noted. First, the current study findings obtained exclusively in children of Northern European ancestry are not necessarily applicable to other ethnicities. Second, only one relevant childhood age range has been examined, and it remains unproven whether adolescents during and at the end of puberty, as well as adults of both sexes, show comparable responses. Third, the 24-h urine samples were retrospectively analyzed. Fourth, since this study was exclusively noninvasive, serum total CO_2_ or bicarbonate measurement data were not available, and it was thus not possible to ascertain whether differences in circulating bicarbonate levels had already been present between the groups. However, as one fifth of the children consuming low FV exhibited 24-h urine pH values below 5.5, it can be assumed that at least some of them had serum bicarbonate concentrations near the bicarbonate threshold of 22 mEq/L [69] thus having a subclinical acidosis. Fifth, aldosterone status could only be assessed indirectly by aldosterone precursors as direct aldosterone metabolites were not assessed in the urinary steroid profile analysis. Sixth, the specifically cross-sectional study design does not allow conclusions about the long-term effects of the respective habitual dietary intakes and about potential changes in the trajectories of glucocorticoid responses to given acid load stimuli.

In conclusion, our quasi-experimental study in healthy 6–10 years old children on moderately high protein intakes demonstrates that a low FV-intake in the range of around 200 g/day or below can go hand in hand with an increased glucocorticoid activity and an increased cortisol secretion when compared to children eating abundant FV. The close relationship, for the first time shown between renal ammonium excretion and specific renally metabolically relevant glucocorticoids, convincingly illustrates the physiological interdependency of the kidneys’ ammoniagenesis and cortisol availability. Thus, to minimize along with certain nutrient deficits also the intrarenal stressor levels of high ammonia concentrations and unnecessarily high cortisol levels in children, an appropriately high FV-intake should be a central nutritional goal for all children. Given the very probable adverse long-term effects of intrarenally high ammonium concentrations and of even only mildly elevated cortisol levels on renal, metabolic, cardiovascular, mental, and bone health, there should be an imperative for all involved in nutrition-politics and -education, to foster children’s availability of and appetite for abundant FV.

## Supplementary Information

Below is the link to the electronic supplementary material.

ESM1 (DOCX 15.2 KB)

## Data Availability

Data described in the manuscript will be made available upon request pending application to and approval by the Institute of Nutrition and Food Sciences, Nutritional Epidemiology, University of Bonn, Bonn, Germany.

## Declarations

### Data sharing statement

Data described in the manuscript will be made available upon request pending application to and approval by the Institute of Nutrition and Food Sciences, Nutritional Epidemiology, University of Bonn, Bonn, Germany.
