# Supplementary material for: Low fruit and vegetable intake in children: a dietary stressor raising renal ammonium and adrenal cortisol secretion
Source: Pflugers Arch. 2026 Mar 23;478(3):30. doi: 10.1007/s00424-026-03162-3 (PMC13006471; doi:10.1007/s00424-026-03162-3)
Supplement: Supplementary file 1 — (DOCX 15.2 KB) [file 424_2026_3162_MOESM1_ESM.docx]

| **Supplemental Table 1 Regressions of 24-h urinary glucocorticoids on fruit and vegetable (FV) intake** | | | | |
| --- | --- | --- | --- | --- |
|  | **β-values** | **95% CI** | ***P*** | **R^2^_model_** |
| **Urinary free cortisol** |  |  |  |  |
| FV | -0.21 | -0.41, -0.0045 | 0.04 | 0.17 |
| Sodium | 1.01 | 0.52, 1.49 | < 0.0001 |  |
| Organic acid | 1.11 | 0.34, 1.88 | 0.005 |  |
|  |  |  |  |  |
| **Urinary free cortisone** |  |  |  |  |
| FV | -0.054 | -0.25, 0.14 | 0.5 | 0.23 |
| Sodium | 0.53 | 0.04, 1.03 | 0.04 |  |
| Nitrogen | 2.05 | 1.18, 2.91 | < 0.0001 |  |
| Duration of storage | -0.63 | -1.04, -0.22 | 0.003 |  |
|  |  |  |  |  |
| **Urinary 20α-dihydrocortisol^a^** |  |  |  |  |
| FV | -0.07 | -0.14, 0.006 | 0.07 | 0.16 |
| Body surface area | 0.45 | 0.30, 0.61 | < 0.0001 |  |
| R^2^_model_, total explained variance. | | | | |
| ^a^Missing values of 20α-dihydrocortisol were imputed using its limit of detection (LOD). | | | | |
